# Neuroanatomy in mouse models of Rett syndrome is related to the severity of Mecp2 mutation and behavioral phenotypes

**DOI:** 10.1186/s13229-017-0138-8

**Published:** 2017-06-26

**Authors:** Rylan Allemang-Grand, Jacob Ellegood, Leigh Spencer Noakes, Julie Ruston, Monica Justice, Brian J. Nieman, Jason P. Lerch

**Affiliations:** 1 Mouse Imaging Centre, 25 Orde Street, Toronto, M5T 3H7 Ontario Canada; 2 Neurosciences and Mental Health, Hospital for Sick Children, 555 University Ave, Toronto, M5G 1X8 Ontario Canada; 3 Genetics and Genome Biology, Hospital for Sick Children, 555 University Ave, Toronto, M5G 1X8 Ontario Canada; 4 Physiology and Experimental Medicine, Hospital for Sick Children, 555 University Ave, Toronto, M5G 1X8 Ontario Canada; 5 Department of Medical Biophysics, Faculty of Medicine, University of Toronto, 101 College Street, Suite 15-701, Toronto, M5G 1L7 Ontario Canada; 6 Ontario Institute of Cancer Research, 661 University Ave, Toronto, Suite 510, M5G 0A3 Ontario Canada

**Keywords:** Magnetic resonance imaging, Rett syndrome, Mecp2 mouse models, Neuroanatomy

## Abstract

**Background:**

Rett syndrome (RTT) is a neurodevelopmental disorder that predominantly affects girls. The majority of RTT cases are caused by de novo mutations in methyl-CpG-binding protein 2 (MECP2), and several mouse models have been created to further understand the disorder. In the current literature, many studies have focused their analyses on the behavioral abnormalities and cellular and molecular impairments that arise from Mecp2 mutations. However, limited efforts have been placed on understanding how Mecp2 mutations disrupt the neuroanatomy and networks of the brain.

**Methods:**

In this study, we examined the neuroanatomy of male and female mice from the Mecp2^tm1Hzo^, Mecp2^tm1.1Bird/J^, and Mecp2^tm2Bird/J^ mouse lines using high-resolution magnetic resonance imaging (MRI) paired with deformation-based morphometry to determine the brain regions susceptible to Mecp2 disruptions.

**Results:**

We found that many cortical and subcortical regions were reduced in volume within the brains of mutant mice regardless of mutation type, highlighting regions that are susceptible to Mecp2 disruptions. We also found that the volume within these regions correlated with behavioral metrics. Conversely, regions of the cerebellum were differentially affected by the type of mutation, showing an increase in volume in the mutant Mecp2^tm1Hzo^ brain relative to controls and a decrease in the Mecp2^tm1.1Bird/J^ and Mecp2^tm2Bird/J^ lines.

**Conclusions:**

Our findings demonstrate that the direction and magnitude of the neuroanatomical differences between control and mutant mice carrying Mecp2 mutations are driven by the severity of the mutation and the stage of behavioral impairments.

## Background

Rett syndrome (RTT) is a neurodevelopmental disorder caused, in over 90% of cases, by sporadic mutations in the X-linked gene, methyl-CpG-binding protein 2 (*MECP2*) [1]. Although boys carrying *MECP2* mutations do not typically survive infancy, girls experience a period of normal development between 6 and 18 months of age, followed by a decline in fine motor coordination skills, speech, autonomic irregularities, cognitive abilities, and stereotypic hand movements [2–5]. These behavioral impairments have been associated with an overall decrease in brain weight along with structural disruptions at the cellular level, such as decreased dendritic length, reduce spine density and cell body size that have been observed post-humous in female RTT patients [6–8]. However, the type and location of *MECP2* mutation influence the severity of these phenotypes, with disruptions within the functional domains of *MECP2* as well as mutations near the N terminus leading to more detrimental outcomes than mutations that occur near the C terminus [9–11].

To further understand RTT, mouse models have been engineered to tease apart how *Mecp2* disruption affects the brain and behavior. Similarly to humans, Mecp2-null mice experience a period of normal development followed by a loss of motor control, disrupted autonomic regulation and impaired learning and memory [12, 13]. Further investigation into the biology of these models has highlighted the importance of *Mecp2* within the brain where it mediates transcription through epigenetic modifications to the chromatin structure [14]. Mice that lack functional *Mecp2* have fewer and weaker excitatory synaptic connections [15, 16] and impaired synaptic plasticity [13, 17, 18]. Additionally, loss of *Mecp2* within the norepinephrine [19], dopaminergic, and serotonergic [20] neurotransmitter systems and within the hypothalamic [21], interneuronal [22, 23], and astroglia [24, 25] cellular populations leads to circuit-specific impairments in the brain and behavior. Along with the heterogeneity of cellular and molecular phenotypes, region-specific differences in excitability have been found across the brain [16, 26]. The culmination of these findings suggest that rather than targeting a specific cell type or neurotransmitter system, *Mecp2* plays a global role that is integral to normal brain structure and function.

Continued efforts into understanding the pathophysiology caused by *Mecp2* disruption requires emphasis on system-wide metrics in order to identify key nodes and networks of susceptibility. Magnetic resonance imaging (MRI) has the ability to acquire high-resolution, neuroanatomical information across the mouse brain [27–29]. Additionally, the high spatial resolution of MRI and the statistical analyses applied to the acquired images has previously been used to phenotype structural differences in mouse models of neurodegenerative and developmental disorders [30–34] and is sensitive to subtle changes in neuroanatomy following training on learning and memory paradigms [35, 36]. Thus, MRI is an important tool for localizing and quantifying anatomical differences across the brain and may reveal important insights into RTT when used in mouse models with *Mecp2* mutations.

Previous MRI studies of human RTT patients have identified volume loss in frontal gray matter, basal ganglia, substantia nigra, midbrain, cerebellum, and brainstem [37–39]. MRI studies of Mecp2-null mice also show volume reductions in many of the same regions as humans, suggesting that these models recapitulate the gross anatomical impairments of the human phenotype [40, 41]. However, these studies limited their analyses to a few regions of interest only providing a snapshot of the profile of volumetric changes within the *Mecp2* disrupted brain.

To overcome this limitation, we examined male and female mice from three Mecp2 mouse models using whole-brain anatomical MRI sequences paired with deformation-based morphometry to identify the regions of the brain affected by *Mecp2* disruption. To determine how *Mecp2* mutation severity affects brain structure, we included Mecp2 mouse models with severe phenotypes caused by silencing of the *Mecp2* gene by the complete removal of exon 3 / 4 (Mecp2^tm1.1Bird/J^[42]) and via a STOP-neomycin cassette within intron 2 (Mecp2^tm2Bird/J^[18]) with mice possessing less severe phenotypes driven by a truncation mutation at amino acid 308 which eliminates the C-terminal end of *Mecp2* (Mecp2^tm1Hzo^[43]). Specifically, for the Mecp2^tm1Hzo^ line, we tested the effect of the mutation in four separate experimental groups; 60-day-old hemizygous males (Mecp2^308/y[B6,P60]^) and 200-day-old hemizygous males (Mecp2^308/y[B6,P200]^), heterozygous (Mecp2^308/x[B6,P200]^), and homozygous (Mecp2^308/308[B6,P200]^) females and compared them to their respective wild-type controls. For the Mecp2-null lines, 60-day-old Mecp2^tm1.1Bird/J^ (Mecp2^NULL/y[129,P60]^) mice along with Mecp2^tm2Bird/J^ hemizygous males on a C57BL/6J (Mecp2^STOP/y[B6,P60]^) and hybrid C57/CBA (Mecp2^STOP/y[Hyb,P60]^) background were compared to controls along with 200-day-old Mecp2^tm2Bird/J^ heterozygous females (Mecp2^STOP/x[B6,P200]^) (Table 1). In addition to the comparison between mutant and wild-type mice, we also explored the relationship between the progression of RTT-related phenotypic impairments and the neuroanatomy to further understand the link between brain structure and behavior. Our findings demonstrate that the structure of the mouse brain is dependent on *Mecp2* in adulthood, with variability in neuroanatomical outcomes driven by the type of the mutation as well as the severity of phenotypic impairments.

**Table 1:** Mouse demographics and experimental parameters used to analyzed neuroanatomy in each experimental group

Exp. group	Allele	Background	Sex	Age	WT	Mut	Imaging
Mecp2^308 /y[B6,P200]^	Mecp2^tm1Hzo^	C57BL/6J	M	200	9	10	100G/cm,TR=325ms,TEeff=40ms,32 *μ*m
Mecp2^308 /x[B6,P200]^	Mecp2^tm1Hzo^	C57BL/6J	F	200	11	12	100G/cm,TR=325ms,TEeff=40ms,32 *μ*m
Mecp2^308 /308[B6,P200]^	Mecp2^tm1Hzo^	C57BL/6J	F	200	11	11	100G/cm,TR=325ms,TEeff=40ms,32 *μ*m
Mecp2^308 /y[B6,P60]^	Mecp2^tm1Hzo^	C57BL/6J	M	60	9	10	12G/cm,TR=2000ms,TEeff=42ms,56 *μ*m
Mecp2^308 /y[B6,P42]^	Mecp2^tm1Hzo^	C57BL/6J	M	42	5	5	Weight calculations
Mecp2^NULL/y[129,P60]^	Mecp2^tm1.1Bird^	129 /SvEvTac	M	60	13	9	30G/cm,TR=350ms,TEeff=30ms,40 *μ*m
Mecp2^STOP/y[B6,P60]^	Mecp2^tm2Bird^	C57BL/6J	M	60	10	13	30G/cm,TR=350ms,TEeff=30ms,40 *μ*m
Mecp2^STOP/x[B6,P200]^	Mecp2^tm2Bird^	C57BL/6J	F	200	11	11	30G/cm,TR=350ms,TEeff=30ms,40 *μ*m
Mecp2^STOP/y[Hyb,P60]^	Mecp2^tm2Bird^	C57 /CBA	M	60	14	15	30G/cm,TR=350ms,TEeff=30ms,40 *μ*m

Mecp2 allele (Mecp2^tm1Hzo^, Mecp2^tm1.1Bird^, Mecp2^tm2Bird^), mouse background (C57BL/6J, 129/SvEvTac, C57/CBA hybrid), sex (male, female), age (days), sample size for WT and mutant mice (mut), gradient strength (G/cm), repetition time (TR), effective echo time (TEeff), and final image resolution (*μ*m) for each experimental group. Experimental groups were named based on the Mecp2 allele, background and age

## Methods

### Experimental animals

In this study, three different Mecp2 mouse lines were used for experimentation: B6.129P2-Mecp2^tm2Bird/J^ (Mecp2^tm2Bird/J^) female mice on a C57BL/6J background were purchased from Jackson Laboratory (stock no. 6849) and maintained by breeding to C57BL/6J males. Mecp2^tm2Bird/J^ females were also crossed with CBA.CaJ males obtained from Jackson laboratories (stock no. 0654) to establish a C57/CBA hybrid colony. 129-Mecp2^tm1.1Bird/Jus^ mice on a 129S6/SvEv background were acquired from the existing active colony at the Toronto Centre for Phenogenomics. B6.129S-Mecp2^tm1Hzo/J^ were acquired from James Ellis’ laboratory at the Hospital for Sick Children (Toronto, ON) or purchased directly from Jackson Laboratory (stock no. 5439). All mice were housed 2–5 per cage and maintained on a 12-hour light/dark cycle with ab libitum access to food and water. The Toronto Centre for Phenogenomics Animal Care Committee approved all experiments.

Experimental groups included Mecp2 mutant (hemi-, hetero- or homozygotes) and matched WT control mice from both sexes that were either 60 or approximately 200 days of age. An additional group of 42-day-old hemizygous and WT males from the Mecp2^tm1Hzo^ line were used for weight calculations only. All other experimental groups were used for weight and neuroanatomical analyses. A total of 178 mice were used in this study. The mouse line, background, sex, age, and sample size for each experimental group are summarized in Table 1.

### Scoring Rett-related phenotypes

As previously outlined [18], experimental mice were scored on six symptoms known to arise from Mecp2 disruption: lack of mobility, abnormal gait, presence of a hindlimb clasp, increased tremors, breathing irregularities, and overall deterioration in general condition. These behaviors were scored as absent (0), present (1), or severe (2), and a total score was generated by adding the six scores together. Body weight and phenotype scores were measured on the laboratory bench in the same location and time of day prior to perfusion and imaging.

### Tissue Preparation for MRI

Mice were anesthetized and intracardially perfused as previously described [44]. Briefly, following transcardial perfusion with heparin, 4% paraformaldehyde and 2 mM ProHance (Bracco Diagnostics), heads were decapitated and the skin, lower jaw, ears, and the cartilaginous nose tip were removed. The remaining skull containing the brain was soaked overnight in 4% paraformaldehyde/2 mM ProHance and then transferred to PBS, 0.02% sodium azide and 2 mM Prohance for at least 30 days prior to magnetic resonance imaging (MRI) acquisition [45].

### Image acquisition

Over the time course of the study, major hardware and software upgrades were made to our imaging system in an effort to increase throughput and image resolution. Thus, the anatomical MRI scans for many of the experimental groups were acquired using different imaging hardware (e.g., gradient size and strength) and acquisition/sequence parameters (see Table 1 for the imaging parameters used for each experimental groups). Although the base acquisition and sequence parameters differed between some of the experimental groups, all were scanned in the same multi-channel 7.0-T MRI scanner (Agilent Technologies) with a T2-weighted, fast spin echo sequence to optimize signal to noise and gray to white matter contrast.

At the beginning of the study, anatomical images were acquired in three custom-built solenoid coils within an insert gradient (6-cm inner diameter bore, rise time of 150 *μ*s, maximum gradient strength = 100 g/cm) with the following sequence parameters: Cartesian acquisition of k-space, TR = 325 ms, and TEs = 10 ms per echo for 6 echos with the center of k-space acquired on the 4th echo, 4 averages, field-of-view (FOV) = 14 × 14 × 25 mm^3^, matrix size = 432 × 432 × 780, 32 *μ*m isotropic resolution, total imaging time was 11 h. Images were downsampled to 64 *μ*m prior to analysis [46]. Upgrades to our sequence design and coil array allowed for more than 3 brains to be imaged in parallel, increasing throughput using 16 solenoid coils (30 cm inner diameter bore, maximum gradient strength of 12 g/cm; using the following image sequence parameters: Cartesian acquisition of k-space, TR = 2000 ms, echo train length = 6, TEeff = 42 ms, 2 averages, FOV = 14 × 28 ×−25 mm^3^, matrix size = 250 × 504 × 450, 56 *μ*m isotropic resolution, for total imaging time of 11.7 h) [35]. By the end of the study, a stronger gradient (2 cm inner diameter bore, maximum gradient strength = 30 g/cm) was installed and a new acquisition scheme was used: cylindrical acquisition of k-space, TR = 350 ms, echo spacing = 12 ms for 6 echoes, TEef f= 30 ms, FOV = 20 × 20 × 25 mm^3^, matrix = 504 × 504 × 630, 40 *μ*m isotropic resolution, for a total imaging time of approximately 14 h.

### Image registration

The registration method has previously been outlined in detail [28, 35]. In this study, image registration was used to align all images within an experimental group into the same space such that corresponding anatomical features become superimposed, thereby allowing statistical comparison. After a series of iterative linear and nonlinear registration steps, a consensus average representing each individual brain and the deformations of each image to this average is generated [47–49]. The Jacobian determinant, a metric of expansion or contraction of a voxel, was then extracted from the deformations fields, converted to a logarithmic scale and blurred with a 200 *μ*m FWHM Gaussian smoothing kernel. The resulting blurred Jacobians are then used as a measure of volume within each voxel of the brain and are fed into our statistical analyses as the dependent variable. To determine the volume of the independent anatomical regions, an atlas with 159 segmented regions [50–52] was aligned with each experimental group’s consensus average allowing for the labels to be back-propagated to each individual brain. The unblurred Jacobian determinants are then summed within the segmented labels to generate a measure of volume within each anatomical region.

In this study, six separate registration pipelines were used to align brain images of wild-type and mutant mice from the experimental groups. The first registration was performed by pooling images of male and female mice from the Mecp2^308/y[B6,P200]^, Mecp2^308/x[B6,P200]^, and Mecp2^308/308[B6,P200]^ experimental groups. Wild-type and mutant mice from the remaining experimental groups (Mecp2^308/y[B6,P60]^, Mecp2^NULL/y[129,P60]^, Mecp2^STOP/y[B6,P60]^, Mecp2^STOP/y[Hyb,P60]^, Mecp2^STOP/x[B6,P200]^) were aligned using separate registration pipelines.

### Analysis and statistics

In order to determine the effect of Mecp2 disruption on the neuroanatomy within each experimental group, a linear model was computed within every voxel of the brain relating the fixed effect of genotype (mutant vs. WT) to the absolute Jacobian determinant. In order to determine the direction and magnitude of the effect, the percent difference in volume was calculated between mutant and WT mice within voxels that survived correction for multiple comparisons. The percent difference in volume was also computed across 159 segmented anatomical regions. For these analyses, the group containing the mutant mice (hemizygous, heterozygous, homozygous) was compared to their corresponding controls, thus normalizing the effect within each experimental group.

Voxelwise correlations between neuroanatomical volume and phenotype score were derived using a linear model relating the normalized Jacobian determinant to total phenotype score. In order to determine whether males and females are differentially affected by Mecp2 disruption, a sex (male, female) by Mecp2 status (silent, functional) interaction was computed using the absolute Jacobian determinants as the dependent variable. The silent group contained the hemizygous Mecp2^308 /y[B6,P200]^ males and the homozygous Mecp2^308/308[B6,P200]^ females, both of which lack WT copies of Mecp2, while the functional groups were composed of their matched WT controls. Differences in total brain volume and body weight were computed using the Student’s *t* test. The false discovery rate correction was used to control for multiple comparisons [53]. Statistical analysis were conducted using the RMINC package (https://github.com/Mouse-Imaging-Centre/RMINC) in the R statistical environment (http://www.r-project.org).

## Results

### Neuroanatomical differences in mice possessing the Mecp2^tm1Hzo^ truncation mutation

To determine the effect of the Mecp2^tm1Hzo^ truncation mutation on the brain, we scanned P60 hemizygous and P200 hemizygous, heterozygous, homozygous and matched WT controls with high-resolution MRI and used a voxelwise and regional-based analysis within segmented anatomical regions to determine the location, direction, and magnitude of the neuroanatomical differences.

**Fig. 1:**
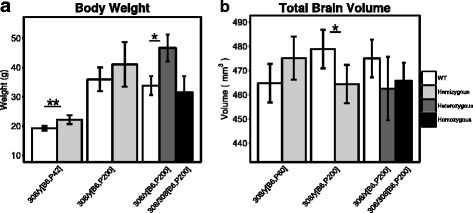
Differential effect of mutation on body weight and total brain volume of male and female Mecp2^tm1Hzo^ mice. **a** Body weight (grams) and **b** total brain volume (mm^3^) is shown for 200-day-old WT (*white bar*) along with hemizygous (Mecp2^308/*y*[B6,P200]^, *light gray*), heterozygous (Mecp2^308/*x*[B6,P200]^, *medium gray*), or homozygous (Mecp2^308/308[B6,P200]^, *dark gray*) mice carrying the Mecp2^tm1Hzo^ allele. In order to assess the effect of the Mecp2^tm1Hzo^ allele during early adulthood, body weight measurements were collected from 42-day-old WT and hemizygous (Mecp2^308/*y*[B6,P42]^) males, while total brain volume was measured from 60-day-old WT and hemizygous (Mecp2^308/*y*[B6,P60]^) males. All mice were on a C57BL/6J background (B6). Each mutant mouse was compared to their WT matched control using a *t*test with a false discovery rate threshold to control for multiple comparisons. *q < 0.05, **q < 0.01

The Mecp2^tm1Hzo^ truncation mutation differentially affected body weight (Fig. 1 a) and total brain volume (Fig. 1 b) across experimental groups. Young hemizygous males were significantly heavier (Mecp2^308 /*y*[B6,P42]^, 13%, *t*= −4.93, *q* < 0.01) and had a 2.2% larger total brain volume compared to WT at P60. Conversely, P200 heterozygous females were significantly heavier than WT (Mecp2^308 /*x*[B6,P200]^, >27%, *t* = −2.43, *q* < 0.05) with a 2% smaller total brain volume than controls. Although the P200 hemizygous and homozygous mutants also had 2–3% smaller brains compared to controls, this difference was only significant in the Mecp2^308 /*y*[B6,P200]^ group (*t* = 2.92, *q* < 0.05).

**Fig. 2:**
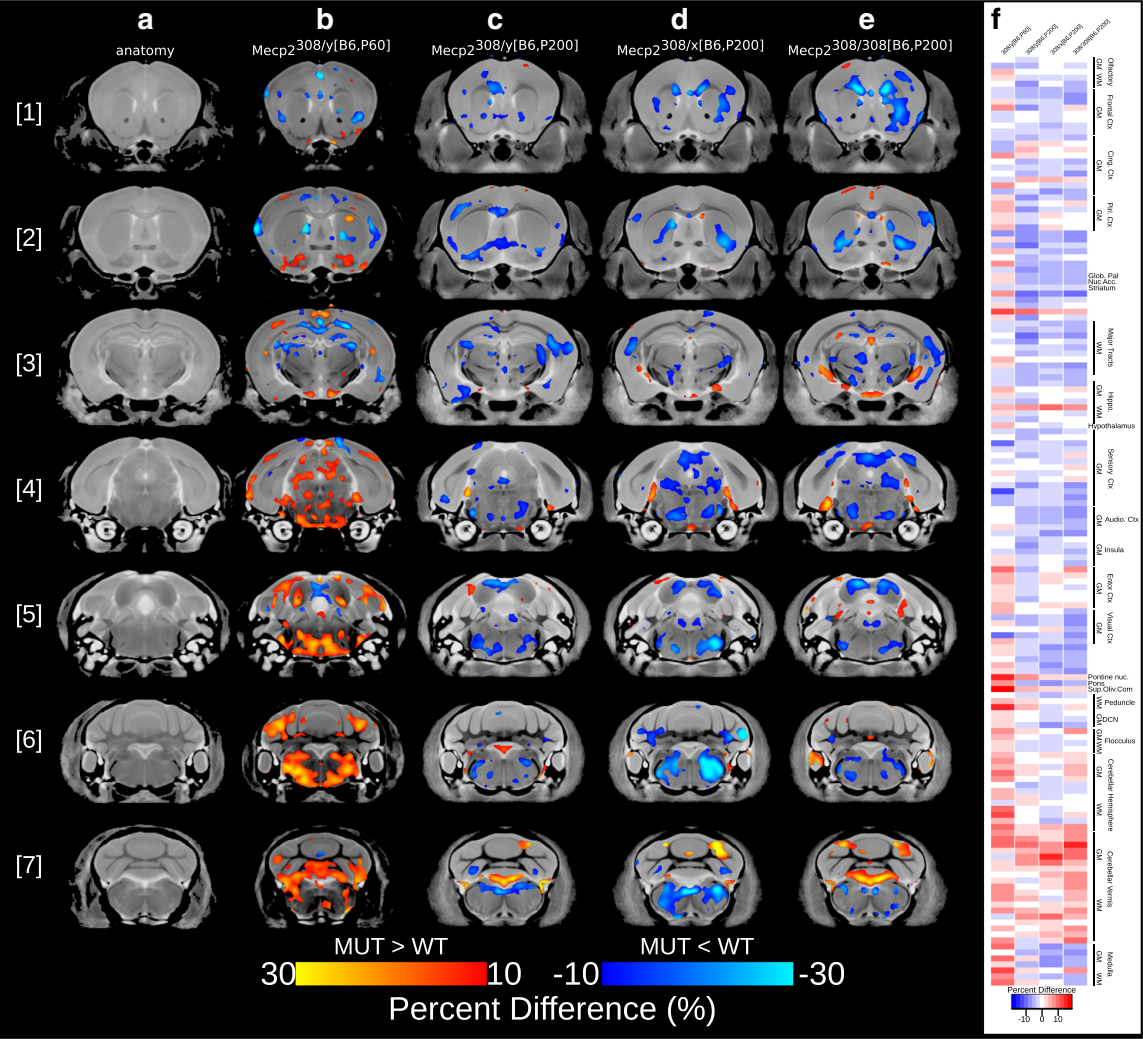
Neuroanatomical differences between mutant and WT mice carrying the Mecp2^tm1Hzo^ truncation mutation. Neuroanatomy was analyzed on a voxelwise level across the brain and within 159 segmented anatomical regions. Coronal slices from the rostral [1] to caudal [7] partitions from the final nonlinear average depicting the neuroanatomy are shown in (**a**). Voxelwise differences between mutant and WT were overlaid on the final nonlinear average for the following experimental groups: **b** Mecp2^308/*y*[B6,P60]^; *t* = 2.71, 10% FDR-corrected, **c** Mecp2^308/*y*[B6,P200]^; uncorrected *t* = 2.52, *p* < 0.01, **d** Mecp2^308/*x*[B6,P200]^; *t* = 2.77, 10% FDR-corrected and **e**. Mecp2^308/308[B6,P200]^;*t* = 2.64, 10% FDR-corrected. The magnitude of the effect is shown as a percent difference; Mutants > WT (*red to yellow*), mutants < WT (*dark blue to light blue*). Percent difference in volume across the 159 segmented structures are shown in (**f**). *White bars*indicate no difference, *blue*indicating a smaller volume in mutants compared to WT, and *red*indicating a larger volume in mutants relative to WT

Significant voxelwise differences between P60 Mecp2^tm1Hzo^ and WT males (Mecp2^308/y[B6,P60]^) are shown in Fig. 2 b (10% FDR-corrected). The direction and magnitude of the effect is shown as a percent difference between the two genotypes and was also calculated across the segmented anatomical regions (Fig. 2 f). Regional voxelwise differences between Mecp2^tm1Hzo^ mutants and WT were found at P60 within the medulla [Fig. 2 b - slice 6, 7], which was 11.6% larger when measured across the entire anatomical volume. Mecp2^tm1Hzo^ mutants also had volumetric increases compared to WT across many regions of the cerebellum, such as the uvula lobule (10.3%), paramedian lobule (7.07%), copula pyramis (6.64%) [Fig. 2 b - slice 7], Crus 1 ansiform lobe 6 (9.62%), Crus 2 ansiform lobe 7 (4.27%), simple lobule-lobe 6 (9.3%), and the inferior cerebellar peduncle (16.8%) [Fig. 2 b - slice 6]. Volumetric increases were also localized to the primary (6.02%) and mediolateral secondary (10.9%) visual cortex, inferior colliculus (3.65%), pons (9.26%), superior olivary complex (17.4%) [Fig. 2 b - slices 4,5], and the basal forebrain (7.23%) [Fig. 2 b - slice 2]. Anterior regions of the brain, such as the corpus callosum (3.5%), fimbria (2.7%), dentate gyrus of the hippocampus (2%) [Fig. 2 b - slice 3], striatum (2.73%) [slice 2], secondary motor (2%) and somatosensory (2.43%) cortices [Fig. 2 b - slice 2], and cingulate cortex within the area 32 (4.6%) and 24a (3.9%) delineations [Fig. 2 b - slice 1] were smaller in young Mecp2^tm1Hzo^ mutants compared to controls.

In a similar pattern as the P60 males, P200 Mecp2^308/*y*[B6,P200]^ mutant males were 3–6% smaller within the cingulate cortex area 32, primary somatosensory cortex, and corpus callosum [slice 2] (uncorrected *t* = 2.52, *p* < 0.01, (Fig. 2 c)). However, unlike the P60 males, P200 hemizygous mutants showed only a few volumetric increases within the cerebellum, specifically crus 1 ansiform lobe 6 and uvula lobule [Fig. 2 c - slice 7]. Interestingly, many more volumetric decreases were observed in the P200 hemizygous brain, particularly within the anterior commissure (9%) [Fig. 2 c - slice 2], pons (4.4%), inferior colliculus (2.9%) [Fig. 2 c - slices 4,5], and the medulla (3%) [Fig. 2 c - slices 6,7].

Mecp2^308/*x*[B6,P200]^ and Mecp2^308/308[B6,P200]^ females at P200 showed a similar neuroanatomical phenotype as Mecp2^308/*y*[B6,P200]^ males at the same age, with 3.5– 8.5% volume reductions detected within the cingulate and piriform cortices, striatum, dentate gyrus of hippocampus, pons, and medulla, and volume increases across the lobes of the cerebellum (Mecp2^308/*x*[B6,P200]^: *t* = 2.77; Mecp2^308/308[B6,P200]^: *t* = 2.64, 10% FDR, Fig. 2 d, e). Although the neuroanatomical pattern was similar between heterozygous and homozygous females across most regions of the brain, they differed within regions of the frontal and piriform cortices, nucleus accumbens, primary visual cortex, and the lobules of the cerebellum (uncorrected *t* = 2.51, *p* < 0.01, data not shown). Interestingly, possessing two copies of the truncated Mecp2^tm1Hzo^ gene (i.e., homozygous female mice) exacerbated the neuroanatomical effects leading to a smaller cortex and nucleus accumbens compared to their heterozygous counterparts.

In order to compare any sex differences, a sex by Mecp2 status interaction was run comparing male hemizygous and female homozygous Mecp2^tm1Hzo^ mice. Although this analysis did not survive correction for multiple comparisons, minor localized differences were found in the inferior and superior colliculi at uncorrected thresholds (uncorrected *t* = 2.43, *p* < 0.01).

### Neuroanatomical differences in Mecp2-null models: Mecp2^tm1.1Bird^ and Mecp2^tm2Bird^

**Fig. 3:**
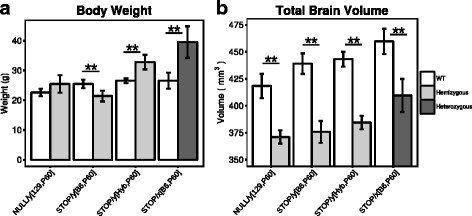
Loss of a functional copy of Mecp2 leads to drastic decreases in total brain volume. **a** Body weight (grams) and **b** total brain volume (mm^3^) measurements from P60 hemizygous males carrying the Mecp2^tm1.1Bird^ (Mecp2^NULL/*y*[129,P60]^) or the Mecp2^tm2Bird^ allele on a C57BL/6J (Mecp2^STOP/*y*[B6,P60]^) or C57/CBA (Mecp2^STOP/*y*[Hyb,P60]^) background. Measurements were also collected from P200 female Mecp2^tm2Bird^ mice on a C57BL/6J background (Mecp2^STOP/*x*[B6,P200]^). Each mutant mouse was compared to their WT matched control using a *t*test with a false discovery rate threshold to control for multiple comparisons. **q < 0.01

In order to further characterize the neuroanatomical phenotypes in the Mecp2 mouse brain, we repeated the experiment using two mouse models of Rett syndrome in which the *Mecp2* gene is completely silent: Mecp2^tm1.1Bird^ and Mecp2^tm2Bird^. Interestingly, the Mecp2^tm2Bird^ gene differentially affects the body weight of mice depending on the background strain and sex, significantly increasing weight in C57/CBA hybrid males (Mecp2^STOP/*y*[Hyb,P60]^, 19.2%, *t* = −−5.43, *q* < 0.01) and Mecp2^STOP/*x*[B6,P200]^ (32%, *t* = −−4.86, *q* < 0.01) females and decreasing weight in Mecp2^STOP/*y*[B6,P60]^ males on a pure C57Bl/6J background by 19.1% relative to controls (*t* = 3.67, *q* < 0.01) (Fig. 3 a). Unlike the P60 Mecp2^308/*y*[B6,P60]^ males, all hemizygous and heterozygous mice with the Mecp2^tm1.1Bird^ or Mecp2^tm2Bird^ mutations had significantly smaller total brain volumes compared to WT, in the range of 10–14% (Mecp2^NULL/*y*[129,P60]^: *t* = 8.12; Mecp2^STOP/*y*[B6,P60]^: *t* = 9.8; Mecp2^STOP/*y*[Hyb,P60])^: *t* = 13.5; Mecp2^STOP/*x*[B6,P60]^:*t* = 5.78; *q* < 0.01) (Fig. 3 b).

**Fig. 4:**
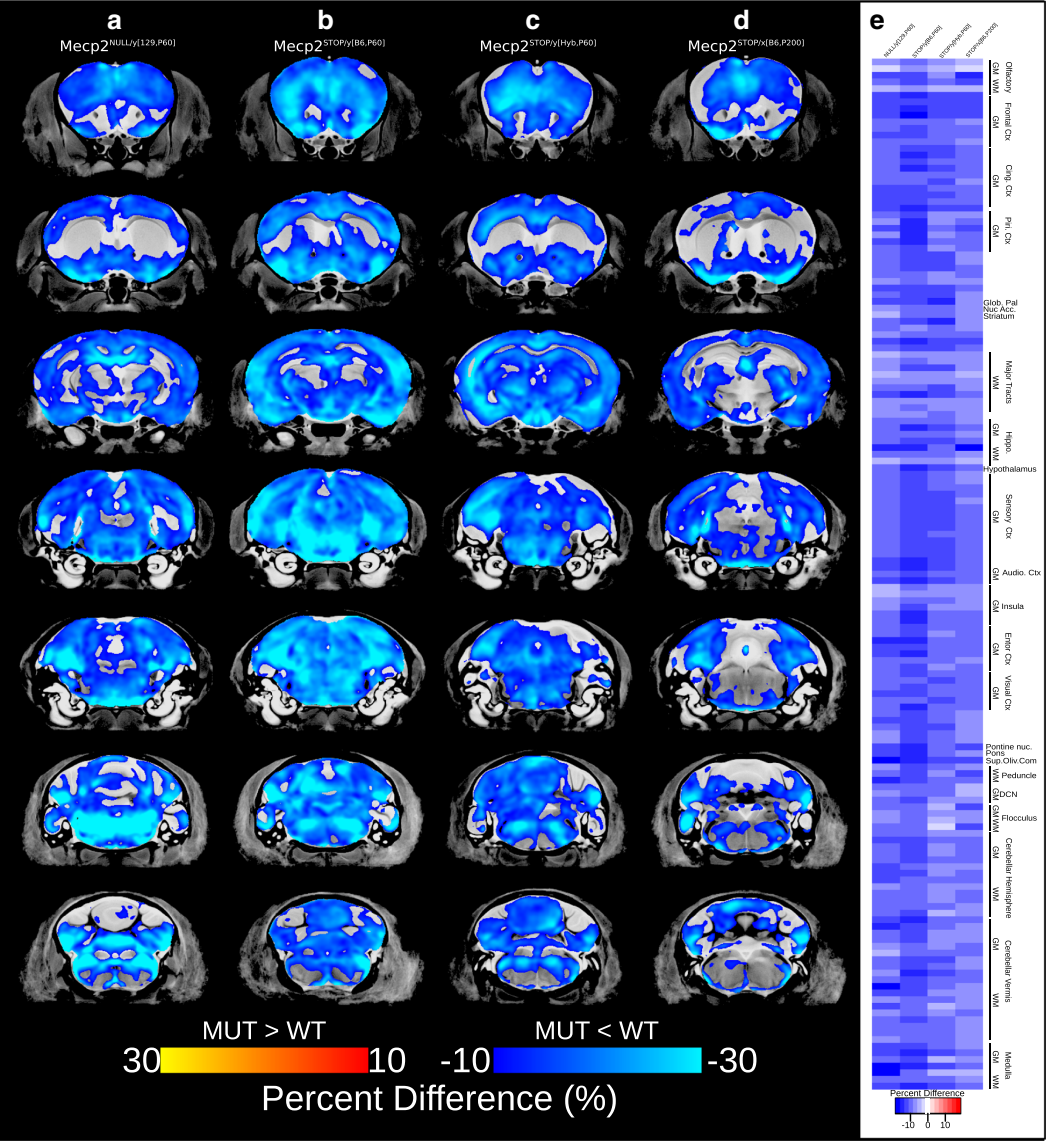
Neuroanatomical differences between mutant and WT mice carrying the Mecp2^tm1.1Bird^ and Mecp2^tm2Bird^ mutations. Significant voxelwise differences (5% FDR-corrected) were overlaid on the final nonlinear average for **a** Mecp2^NULL/*y*[129,P60]^; *t* = 2.15, **b** Mecp2^STOP/*y*[B6,P60]^; *t* = 2.16, **c** Mecp2^STOP/*y*[Hyb,P60]^; *t* = 2.09, and **d** Mecp2^STOP/x[B6,P200]^; t=2.15 experimental groups. The magnitude of the effects is shown as a percent difference; Mutants < WT (*dark blue to light blue*). Percent difference in volume across the 159 segmented structures are shown in (**e**)., with *white bars*indicating no difference and *blue* indicating smaller in mutants compared to WT

Interestingly, significant volumetric decreases relative to WT were found across the hemizygous male and heterozygous female brain in all experimental groups possessing a fully disrupted Mecp2 gene (Mecp2^NULL/*y*[129,P60]^, Fig. 4 a; Mecp2^STOP/*y*[B6,P60]^, Fig. 4 b; Mecp2^STOP/*y*[Hyb,P60]^, Fig. 4 c; *t* = 2.06 –2.16, 5% FDR; Mecp2^STOP/*x*[B6,P60]^, Fig. 4 d; *t* = 2.15, 5% FDR). In all groups, mutants had severe volumetric decreases across the frontal (13–18%), motor (12.9–16.4%), somatosensory (11.4–17.9%), and cingulate (11.1–17.4%) cortices, nucleus accumbens (9.7–12%), ventral striatum (8.75–12.2%), globus pallidus (10–15.7%), midbrain (8.9–13.5%), gray and white matter of the cerebellum (8.3–17.6%), and medulla (8.4–13.46%) (Fig. 4 e).

**Fig. 5:**
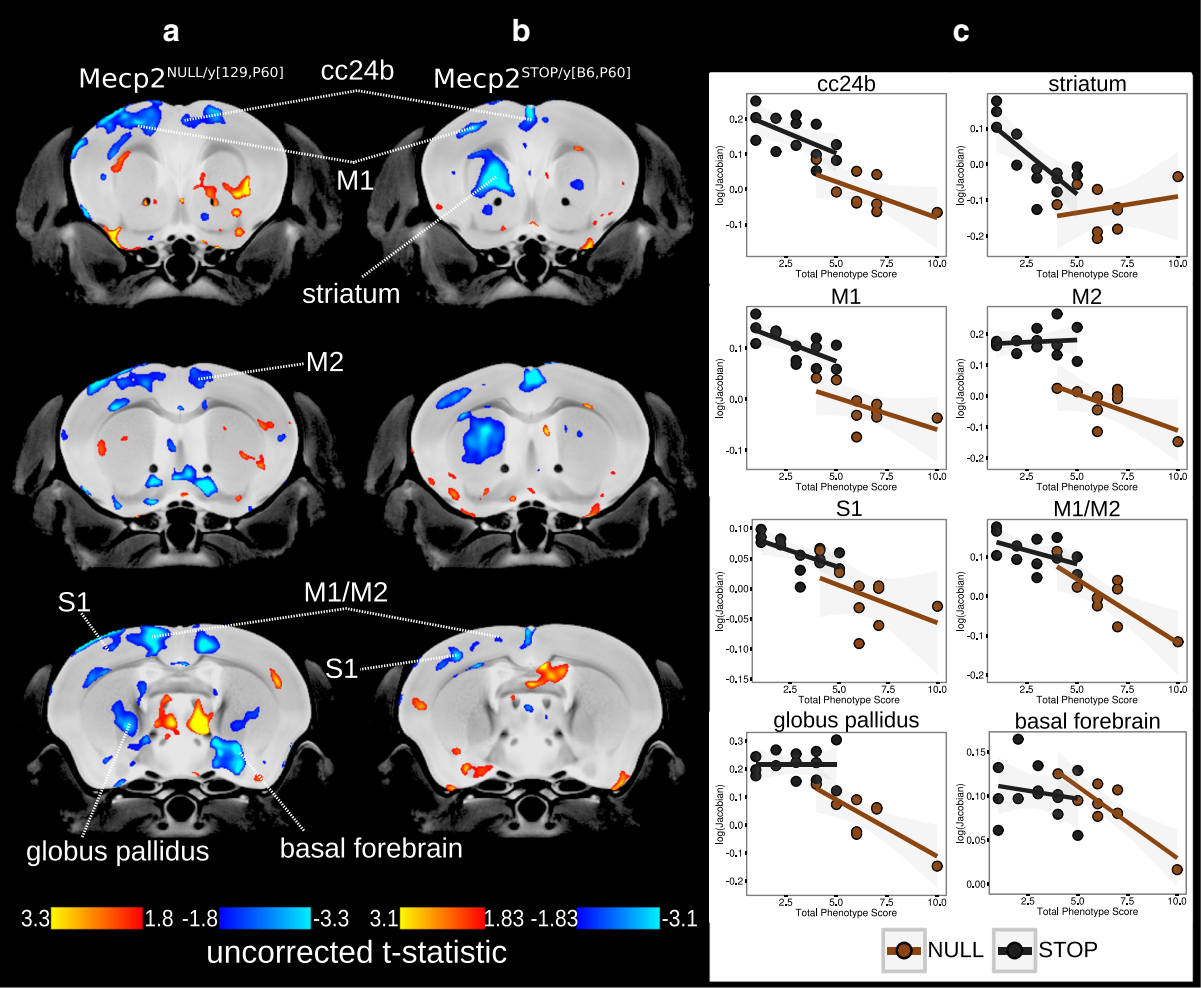
Neuroanatomical correlations with phenotype. Correlations between volume and total phenotype score within the hemizygous **a** Mecp2^NULL/*y*[129,P60]^; uncorrected*t* = 1.8 and **b** Mecp2^STOP/*y*[B6,P60]^; uncorrected = 1.83 brains (uncorrected threshold ranging from *p* < 0.05 to *p* < 0.005). The volume of voxels either negatively or positively correlated with total phenotype score (*blue*and *red*regions, respectively). **c** Plots of the correlation between phenotype score and the normalized Jacobian determinant. Mecp2^STOP/*y*[B6,P60]^) (*black circles and line*), Mecp2^NULL/*y*[129,P60]^ (*brown circles and line*). *cc24b* Cingulate cortex area 24b, *M1* primary motor cortex, *M2* secondary motor cortex, striatum, *S1* primary somatosensory cortex, globus pallidus, basal forebrain

Furthermore, linear trends were found between neuroanatomical volume and phenotype score in both Mecp2^NULL/*y*[129,P60]^ (uncorrected *t* = 1.8, *p* < 0.05, Fig. 5 a) and Mecp2^STOP/*y*[B6,P60]^ (uncorrected *t* = 1.83, *p* < 0.05, Fig. 5 b) hemizygous males. In both experimental groups, negative correlations were found within the cingulate cortex area 24b, primary and secondary motor cortices and primary somatosensory cortex (Fig. 5 c). Additional negative correlations between phenotype score and volume were found in the globus pallidus and basal forebrain in the Mecp2^NULL/*y*[129,P60]^ brain and within the striatum of the Mecp2^STOP/*y*[B6,P60]^ group.

## Discussion

In this study, we imaged the brains of three different Mecp2 mouse models using anatomical MRI parameters paired with deformation-based morphometry to identify the brain regions affected by Mecp2 mutations. Our primary comparison of interest was between the Mecp2^tm1Hzo^ truncation model, that possess a STOP sequence downstream of codon 308 which eliminates the C-terminal of the coding sequence [43], with two models that do not express functional Mecp2 protein, either by the complete removal of exon 3 and 4 (Mecp2^tm1.1Bird^[42]) or via a STOP-neomycin cassette within intron 2 (Mecp2^tm2Bird^[18]).

We reasoned that susceptible brain regions would show a similar neuroanatomical phenotype in mutant mice from all three Mecp2 models. Regardless of mutation type, regional volumes of the frontal, cingulate, sensory, and motor cortices, as well as the striatum, thalamus, and white matter tracts were smaller in mutant mice relative to their WT controls. Interestingly, these neuroanatomical changes recapitulate the findings from human Rett syndrome, highlighting a particular cross-species susceptibility within cortex, caudate putamen, and white matter [37, 38, 54]. However, the foremost difference between experimental groups was the magnitude of the volumetric differences between genotypes. In the Mecp2^tm1Hzo^ group, the majority of neuroanatomical differences between mutants and controls were within the range of 5–10% with isolated peaks of volumetric differences greater than 20% localized within specific brain regions. However, mutants carrying an early occurring truncation mutation that disrupts the functional domains of *Mecp2* had volumetric decreases ranging from 15 to 30% that spanned across all cortical and subcortical areas. Thus, despite similarities in the direction of the volumetric effects within a subset of regions, mutations that ablate the functional domains of *Mecp2* have a more detrimental effect on the brain than the truncation of the C-terminal.

Furthermore, the volume within many of these regions directly related to the severity of phenotypic impairments. P60 Mecp2^NULL/*y*[129,P60]^ and Mecp2^STOP/*y*[B6,P60]^ males with more severe behavioral phenotypic impairments had correspondingly smaller volume within voxels of the striatum, cingulate cortex area 24b and 32, primary and secondary motor cortices, striatum, and globus pallidus (Fig. 5). While many of these findings were bilateral, correlations between neuroanatomy and phenotype score were found to be localized to the left striatum in the Mecp2^STOP/*y*[B6,P60]^ mutant brain. Previous studies have demonstrated that structural volumes within the C57BL/6J mouse brain are asymmetrical within particular cortical and subcortical regions, including the striatum [55]. Although a direct structure/function relationship has not be drawn, the findings suggest that an underlying mechanism exists which may skew the functional capacity within one hemisphere over the other. Furthermore, unilateral structural changes within the striatum have previously been shown to occur following training on a procedure memory task, demonstrating a hemispheric-specific responsiveness to behavioral outcomes [35]. The culmination of these findings provide an explanation for the unilateral correlations within the striatum which may represent a hemispheric-specific susceptibility to increasing phenotype severity in the Mecp2-null brain.

At this time, we are unable to fully understand the nature of the relationship between RTT-related phenotypic impairments and the volumetric changes within corresponding neuroanatomical areas. In order to make sense of the relevance of this relationship, additional biochemical and histological assays would need to be added to the imaging and behavioral data to ascertain its diagnostic or therapeutic potential. Interestingly, the volume of frontal cortical regions have also been shown to be correlated with behavioral impairments in girls with Rett syndrome [39], highlighting that the relationship between volume and behavior is particularly important to further investigate.

The behavioral/volume relationship seen here may explain the difference in magnitude of the neuroanatomical effects between the Mecp2^tm1Hzo^ and Mecp2-null models (Mecp2^tm1.1Bird^ and Mecp2^tm2Bird^). As seen in both humans and mice, late occurring truncations of Mecp2 lead to a less severe progression and overall behavioral outcome than early occurring mutations that ablate the functional domains [9, 10, 42, 43]. With the established connection between phenotype and volume within particular brain regions, the differences in neuroanatomical effects between these models may be a reflection of the deviation in phenotypic progression and severity. However, this phenomena cannot explain the entire profile of neuroanatomical differences between the models because not all regions are correlated with phenotype score.

Additional neuroanatomical deviations may be driven by differences in the underlying cellular environment. Complete loss of Mecp2 within the brain leads to a reduction in dendritic length, spine density and immunoreactive synaptic markers within the motor cortex and hippocampus [15, 56, 57]. Despite a slight reduction in postsynaptic density length and impaired LTP, no obvious histological and dendritic irregularities were found in the Mecp2^tm1Hzo^ brain [13, 43]. Thus, the drastic cellular and structural deficits observed at the neuropil in the Mecp2-null brain compared to the Mecp2^tm1Hzo^ may compound throughout the levels of the neuroanatomy leading to greater volumetric effects at the mesoscopic level measured by MRI. Although histological analyses are required to determine how the underlying cellular disruptions are driving the volume within these regions, previous studies have demonstrated that MRI paired with deformation-based morphometry is sensitive to subtle changes at the neuropil [35].

Many studies of Mecp2 mouse models use males as their experimental animals to avoid phenotypic variability caused by mosaic X-chromosome inactivation in females. However, a particular concern in the field is that too many studies are conducted using males to model a neurodevelopmental disorder that predominantly affects females. In our study, we included heterozygous and homozygous female mice to determine any sex-specific interactions that may lead to differential outcomes on the neuroanatomy. Interestingly, we found that the pattern of neuroanatomical changes is preserved between males and females from both the Mecp2^tm1Hzo^ and Mecp2-null experimental groups when matching for disease progression rather than age. This was seen on a qualitative inspection between Mecp2^tm2Bird^ males and females and a direct statistical comparison between Mecp2^tm1Hzo^ hemizygous males and homozygous females, both of which lack a wild-type copy of *Mecp2*. The lack of an interaction between sex and *Mecp2* status suggests that males can be used to model the neuroanatomical phenotypes caused by *Mecp2* disruptions.

We also found that despite differences in body weight between P60 Mecp2-null male mice, the magnitude and direction of neuroanatomical changes between the two different models without functional *Mecp2* (Mecp2^NULL/*y*[129,P60]^ and Mecp2^STOP/*y*[B6,P60]^), and the two Mecp2^STOP^ models on different backgrounds (Mecp2^STOP/y[B6,P60]^ and Mecp2^STOP/y[Hyb,P60]^) was very similar, highlighting that a separate mechanism drives differences in the neuroanatomy and body weight. These findings suggest that disruption of the functional domains of *Mecp2* leads to a penetrant effect within the brain that transcends differences in genetic background.

Along with finding regions that were similarly affected across Mecp2 models, we were also able to identify brain regions that were differentially affected by the severity of *Mecp2* mutation. Compared to the Mecp2-null groups that show global volumetric reductions, the Mecp2^tm1Hzo^ mutation increased the volume of the mutant cerebellum compared to controls. Additionally, cerebellar volumes were dependent on the amount of functional *Mecp2* alleles, showing a stepwise increase in volume from WT to heterozygous to homozygous females. These findings demonstrate that the severity of *Mecp2* disruption leads to differential effects on the cerebellum.

Unlike the P200 Mecp2^308/*y*[B6,P200]^, Mecp2^308/*x*[B6,P200]^, and Mecp2^308/308[B6,P200]^ experimental groups, the P60 Mecp23^308/*y*[B6,P60]^ hemizygotes are larger than WT in total brain volume and locally across many brain regions. These neuroanatomical differences between P60 and P200 hemizygous males may be related to the stage of pathogenesis, with early stages characterized by mild phenotypes and larger volumes, which later transitions into smaller volumes as the phenotype progresses with age. However, a longitudinal study would be needed to understand the spatial and temporal dynamics of this growth trajectory within the Mecp2^308/*y*^ brain. Overall, these findings are important because they demonstrate that the type of *Mecp2* mutation as well as the stage of pathogenesis can contribute unwanted variance which needs to be controlled when measuring neuroanatomy, particularly in studies that use these outcomes as diagnostic tools or to measure treatment success.

Many of these affected regions are part of neural networks that govern specific behaviors and phenotypes that are impaired by *Mecp2* mutations. One of the hallmark behavioral phenotypes of Rett syndrome is an impairment of motor abilities, manifesting as a loss of purposeful hand movements and coordination in humans [2] and impairments on tasks of motor ability, such as beam walking and rotarod in Mecp2 mouse models [12]. In both species, motor control is governed by the conserved dopaminergic networks that originates in the dopamine-synthesizing cells of the substantia nigra and ventral tegmental area and projects through the striatum, globus pallidus, cortex, and thalamic nuclei [58]. Previous work has shown that *Mecp2* mutations within the substantia nigra and ventral tegmental area leads to the loss of dopamine and dopaminergic metabolites that contributes to the classical parkinsonian features of RTT [20, 59]. Furthermore, selective deletion and reactivation of *Mecp2* within the dopamine-synthesizing neurons of the substantia nigra [60] and the dorsal striatum [61] has demonstrated its importance for psychomotor control. It is perhaps not surprising then that mutant mice from all experimental groups had volume reductions within important nodes of this neural network, such as the primary and secondary motor cortices, striatum, and globus pallidus. Moreover, the volume within these regions was correlated with the progression of Rett-related phenotypes. These findings demonstrate that volume within brain regions that govern motor behaviors are sensitive to *Mecp2* mutations and are tightly related to the stage of behavioral pathogenesis.

In summary, we identified regions of the brain that are similarly affected regardless of mutation severity, sex, and background which are correlated with changes in phenotypic profile. These findings demonstrate that particular regions of the brain are dependent on functional *Mecp2* expression and are thus, most susceptible to Mecp2 disruption.

## Conclusions

As our understanding of the etiology of neurodevelopmental disorders digs deeper into the biology, treatment interventions are becoming more specific, targeting cellular mechanisms and pathways. Although treatment strategies are still in development for RTT, many studies conducted with Mecp2 mouse models have tested a variety of pharmacological and genetic interventions that target the cellular imbalance caused by *Mecp2* disruption [12, 18, 56, 62–64]. The findings from these studies demonstrate that the behavioral and cellular impairments can be rescued highlighting that the brain retains an innate plasticity that can be recruited to restore function in adulthood [65]. However, in order to accurately assay treatment success, noninvasive biomarkers that provide specific information regarding the neurobiological treatment response across the brain are needed. In our study, we demonstrate that MRI paired with deformation-based morphometry is a sensitive method that can detect global changes in the brain influenced by a variety of genetic disruptions and the progression in behavioral phenotypes. Thus, MRI would serve as an important diagnostic tool for assaying the efficacy of treatment interventions on the neuroanatomy, particularly across important neural networks that govern classical RTT symptoms.
